# Editorial to the Special Issue Entitled “Imaging in Immunooncology”

**DOI:** 10.1007/s11307-022-01719-z

**Published:** 2022-03-18

**Authors:** Gilbert O. Fruhwirth, Bettina Weigelin, Heike E. Daldrup-Link, Vladimir Ponomarev

**Affiliations:** 1 Comprehensive Cancer Centre, School of Cancer and Pharmaceutical Studies, King’s College London, London, UK; 2 Department of Preclinical Imaging and Radiopharmacy, Eberhard Karls University, Tübingen, Germany; 3 Cluster of Excellence iFIT (EXC 2180) “Image-Guided and Functionally Instructed Tumor Therapies,”, University of Tuebingen, Tübingen, Germany; 4 Department of Radiology, Stanford University, Stanford, CA USA; 5 Department of Radiology, Memorial Sloan Kettering Cancer Center, New York, NY USA

Cancer immunotherapy is an umbrella term for several different therapy approaches, such as monoclonal antibodies and immune checkpoint inhibitors (ICIs), adoptive T cell transfer therapy, immune system modulators, oncolytic virus therapy, and cancer vaccines. Over the last decade, various new immunotherapy concepts emerged, and several immunotherapies were established for clinical treatments of specific cancers, for example, ipilimumab, a monoclonal antibody targeting CTLA-4 for the treatment of irresectable and/or metastasized melanoma [1] and chimeric antigen receptor-T cell therapies (CAR-T) for the treatment of leukaemia [2]. While these immunotherapeutics have advanced cancer treatment outcomes significantly for distinct patient groups, in some cases even in patients with complete tumour remission, durable responses are generally achieved in only a fraction of the treated patients. Importantly, patient stratification and prediction of treatment success remains a challenging area and it is not *a priori* clear which patients to treat how. Moreover, learning to monitor immunotherapy success adequately has been an area of active research in which significant progress has been made over recent years. To date, tumour volume-based response criteria (Response Evaluation Criteria In Solid Tumours, RECIST; [3]) or PET response criteria in solid tumours (PERCIST) are used to monitor tumour response in many clinical trials. However, RECIST and PERCIST criteria can be complicated in patients undergoing immunotherapy because the influx of immune cells into the tumour environment can lead to a temporary increase in size and/or metabolic activity, causing a ‘pseudoprogression’ of solid tumours. Therefore, more immunotherapy-specific imaging biomarkers of therapy response are urgently needed [4–7]. Immunotherapy toxicities occur when immune cells react against healthy cells and tissues in the body and pose diagnostic as well as management challenges [8]. Therapy resistance phenomena represent another major challenge in immunotherapy [9, 10] and this includes the timely recognition of resistance onset required to trigger treatment alterations. In the context of these challenges, immunotherapy-tailored molecular imaging tools have great potential to support the development of personalized immunotherapy and guide clinicians in their decision-making processes. In this *Special Issue*, we have compiled a set of contributions on imaging cancer immunotherapy with particular relevance to various challenges mentioned above, covering aspects relevant to basic and translational immunotherapy research as well as informing clinical immunotherapy use.

A particularly relevant and still expanding area is concerned with reliably quantifying immunotherapy target presence in solid tumours with the goal to provide a rationale for patient stratification and treatment selection. In this context, many relevant molecular imaging tools exploit the concept of ImmunoPET, which is a diagnostic approach using antibodies or fragments thereof to specifically detect therapy targets *in vivo*. This *Special Issue* contains a comprehensive review on harnessing antibodies for immune cell imaging [11] alongside a preclinical example of a new way of imaging the immune checkpoint receptor TIM3 [12], which has been shown to enhance the antitumour effects of PD-1/PD-L1 ICIs [13]. Antigen-presenting cells express various structurally related proteins on their cell surfaces (B7 class) that regulate immune responses by delivering co-inhibitory or co-stimulatory signals through their receptors. Imaging probes are developed also for B7 family members beyond PD-L1 (i.e. B7-H1) and here, we included an article that exploits targeting ultrasound microbubbles to B7-H3 to non-invasively visualize tumour-draining lymph nodes [14]. Notably, combined ICI treatment is currently developed for various cancer types, whereby treatment order, intervals, and relative dosing regimens remain under investigation. Various molecular imaging tools beyond ImmunoPET are currently applied to this area, and an overview of their use focussed on breast cancer is embedded in this issue [15]. ImmunoPET can also be configured to inform on the immunological state of a tumour by using endogenous immune cells as the imaging target. This can be exploited to enable discrimination of ‘immune hot’ from ‘immune cold’ tumours without a biopsy (cf. [11]). In the quest to better understand the complexities of the immune system’s interaction with the evolving cancer, it cannot be overemphasized how important models are that enable the *in vivo* assessment of immune cell localization, function, and survival. Here, we provide an article by Chawda *et al*. who specifically focus on the application of optical imaging to assess immune cell function in mouse models of cancer [16]. In addition, this *Special Issue* contains a perspective of the state of neutrophil imaging and its potential role in immunooncology [17].

Another emerging potential cancer treatment involving the immune system is, in simple terms, the attempt to exploit ionizing radiation to ‘wake up’ the immune system. Termed ‘abscopal effect’, this was first observed in mice almost 70 years ago [18], however, remained rather silent until the combination of radiotherapy with immunostimulatory agents over the last decade has shown promise for patients suffering from metastatic cancers fuelled by observations that not only irradiated tumours but also distant metastases regressed [19]. As therapies involving ionizing radiation can be accompanied by significant side effects, researchers have repeatedly asked whether similar results might also be obtained using different lower-energy regions of the electromagnetic spectrum, whereby rather conflicting results have been reported, not least because of wildly differing experimental conditions. Hence, the question whether, for example, ultrasound can alter the immune reaction of peripheral solid tumours in humans and animals compared to control conditions without ultrasound application is not straightforward to extract from existing literature. Here, Rix *et al*. provide a systematic review protocol to address this issue. Notably, they extract relevant information from the wealth of existing ultrasound-focussed literature with the aim to enable relevant meta-analyses for this specific research question [20].

Moreover, there is the large arena of vaccination approaches to treat cancer, which can broadly be classified into preventive and therapeutic vaccines, whereby the latter comprise molecular, viral, and cell-based approaches. Tumour antigen-presenting dendritic cells (DCs) as cancer vaccines have been clinically introduced about two decades ago. While they initially didn’t live up to their promise, *in vivo* tracking of DCs to use lymph node presence of the administered DCs as an imaging biomarker to predict efficacy may aid clinical treatment management. In this *Special Issue*, Bulte and Shakeri-Zadeh provide an overview where the field of DC tracking by MRI currently stands as well as a clinical perspective thereof [21]. Notably, the application of fluorine MRI to *in vivo* track DCs is not restricted to immunooncology and is emerging as a valuable tool to study other pathologies. An example of bone marrow-derived DC imaging in the context of pancreatic inflammation is also included here [22].

Among the various immunotherapy concepts, adoptive anti-cancer cell therapies stand out as they involve the administration of living cells. Therapeutic cells are either generated from the patient (autologous) or from a different donor (allogeneic) and *ex vivo* expanded before administration and may or may not be *ex vivo* genetically engineered. In immunooncology, CAR-T cells have been adopted clinically first, and represent a genetically engineered class of autologous therapies. CAR-T cell therapies in clinical practice are primarily intended to treat haematological malignancies. The latest addition to the repertoire approved by The Food and Drug Administration is ciltacabtagene autoleucel for the treatment of adult patients with relapsed or refractory multiple myeloma after four or more prior lines of therapy [23]. It is important to recognize that this class of therapeutics is fundamentally different from molecular therapeutics in that they are living cells, which can vary in number, distribution, and activity *in vivo*. In solid tumours, the efficacy of CAR-T cells is linked to their presence at the tumour site and their survival after administration (unlike medicinal signalling cells that are suggested to exploit the ‘paracrine effect’ cf. [24, 25]). Such adoptively transferred anti-cancer CAR-T cells can directly interact with cancer cells in the case of blood cancers where CAR-T is now routinely, hence spatiotemporal imaging was not regarded key to their development and translation. But in solid tumours, a lack of information about the penetration and survival of CAR-T cells in solid tumours has become a major obstacle for cell therapy development and translation. Molecular imaging can potentially provide information about both cell therapy *in vivo* location and survival. Fundamental immune cell tracking concepts have been reviewed elsewhere [26], while in this *Special Issue*, Sato *et al*. specifically focus on the development of *ex vivo* cell therapy labelling for positron emission tomography (PET)-afforded cell tracking; additionally, they provide insight for labelling different cell types as well as a clinical cell tracking perspective [27]. While radionuclide imaging approaches impress with excellent sensitivity and clinical compatibility, they require an additional imaging test for many patients, who otherwise undergo cancer therapy monitoring with CT or MRI. Cell tracking technologies that utilize MRI for the *in vivo* tumour accumulation of adoptively transferred cell therapies could be more easily integrated in many currently established clinical workflows. Dubois *et al*. report in this *Special Issue* on how to approach this using fluorine-19 MRI on a 3T scanner [28].

The access to reliable information on *in vivo* survival of adoptive cell therapies after administration, however, is more challenging to acquire than their *in vivo* locations and, notably, not accessible employing direct *ex vivo* cell labelling methodology [26]. An elegant approach to provide such *in vivo* survival information is the utilization of gene reporters. This has been exploited preclinically in many different configurations spanning several imaging modalities whereby radionuclide approaches have traditionally dominated due to their excellent sensitivities (cf. [29] for an example on CAR-T persistence in tumours). In this *Special Issue*, Shalaby *et al*. provide preclinical data on their new combined radionuclide-MRI approach that is based on gene reporters for both modalities [30], which might also enable relative sensitivity comparisons in the future.

Moreover, the non-invasive *in vivo* assessment of immune cell function is becoming increasingly important while compared to imaging of immune cell location and survival requires more refined approaches, which are reviewed here by Chawda *et al*. [16]. The concept of exploiting gene reporters for imaging adoptively transferred therapeutic immune cells has already been demonstrated clinically [31], albeit using a foreign reporter in a very special late-stage glioma setting. In recent years, however, significant advances have been made to introduce human PET reporter genes that overcome concerns related to reporter immunogenicity [26]. While gene reporters currently appear clinically only viable to be co-engineered into cell therapies that already require genetic engineering for their efficacy (e.g. CAR-T, CAR-NK, or TCR-T), new translatable imaging concepts are warranted to address important challenges of obtaining cell survival and function information non-invasively on the clinical level.

Molecular imaging has been coming of age, but it continues through new and refined technologies and ever more reliable and robust methodologies to expand and actively fuel significant advancements in immunooncology and immunology. Molecular imaging continues to progress both basic and translational settings and will help unlock new immunotherapy treatment paradigms and aid their translation into clinical practice.
